# New Mastoparan Peptides in the Venom of the Solitary Eumenine Wasp *Eumenes micado*

**DOI:** 10.3390/toxins11030155

**Published:** 2019-03-10

**Authors:** Katsuhiro Konno, Kohei Kazuma, Marisa Rangel, Joacir Stolarz-de-Oliveira, Renato Fontana, Marii Kawano, Hiroyuki Fuchino, Izumi Hide, Tadashi Yasuhara, Yoshihiro Nakata

**Affiliations:** 1 Institute of Natural Medicine, University of Toyama, Toyama, Toyama 930-0194, Japan; cokazuma@kumamoto-u.ac.jp; 2 Immunopathology Laboratory, Butantan Institute, Sao Paulo SP 05503-900, Brazil; marisarangel2112@gmail.com or mrangel@usp.br; 3 Laboratory of Physiology and Animal Toxins, Federal University of West Pará, Santarém PA 68040-070, Brazil; Joacir.ufopa@gmail.com; 4 Department of Biological Sciences, State University of Santa Cruz, Ilhéus BA 45662-900, Brazil; rfontana@uesc.br; 5 Research Center for Medicinal Plant Resources, National Institutes of Biomedical Innovation, Health and Nutrition, Tsukuba, Ibaraki 305-0843, Japan; marii@nibiohn.go.jp (M.K.); fuchino@nibiohn.go.jp (H.F.); 6 Department of Molecular and Pharmacological Neuroscience, Graduate School of Biomedical and Health Sciences, Hiroshima University, Hiroshima, Hiroshima 734-8551, Japan; ihide@hiroshima-u.ac.jp; 7 Laboratory of Microbial Chemistry, School of Pharmacy, Kitasato University, Minato-ku, Tokyo 108-8641, Japan; yasuharat@pharm.kitasato-u.ac.jp; 8 Department of Pharmacology, Hiroshima University Graduate School of Biomedical & Health Sciences, Hiroshima 734-8553, Japan; ynakata@hiroshima-u.ac.jp

**Keywords:** solitary wasp, venom, mastoparan peptide, linear cationic α-helical peptide, amphipathic α-helix structure.

## Abstract

Comprehensive LC-MS and MS/MS analysis of the crude venom extract from the solitary eumenine wasp *Eumenes micado* revealed the component profile of this venom mostly consisted of small peptides. The major peptide components, eumenine mastoparan-EM1 (EMP-EM1: LKLMGIVKKVLGAL-NH_2_) and eumenine mastoparan-EM2 (EMP-EM2: LKLLGIVKKVLGAI-NH_2_), were purified and characterized by the conventional method. The sequences of these new peptides are homologous to mastoparans, the mast cell degranulating peptides from social wasp venoms; they are 14 amino acid residues in length, rich in hydrophobic and basic amino acids, and C-terminal amidated. Accordingly, these new peptides can belong to mastoparan peptides (in other words, linear cationic α-helical peptides). Indeed, the CD spectra of these new peptides showed predominantly α-helix conformation in TFE and SDS. In biological evaluation, both peptides exhibited potent antibacterial activity, moderate degranulation activity from rat peritoneal mast cells, and significant leishmanicidal activity, while they showed virtually no hemolytic activity on human or mouse erythrocytes. These results indicated that EMP-EM peptides rather strongly associated with bacterial cell membranes rather than mammalian cell membranes.

## 1. Introduction

Mastoparan was first isolated from the venom of the vespid wasp Paravespula lewisii as a mast cell degranulating and histamine-releasing principle [1]. Since then, a number of closely related peptides have been found in a variety of social wasp venoms (hornets and paper wasps) [2,3], and they are collectively called mastoparans or mastoparan peptides. The mastoparans are 14 amino acids in length with the C-terminus amidated, and rich in hydrophobic and basic amino acids, which leads to amphipathic chemical character, adopting α-helical secondary structure under proper conditions. This chemical feature is essential for their biological activities associated with the cell membrane, showing histamine releasing from mast cells, and antimicrobial and hemolytic activities. Due to these biological properties, mastoparans have been developed not only for pharmacological research tools but also for therapeutic use, in particular, as a new type of antimicrobial agents [4,5,6].

In our survey of bioactive substances in solitary wasp venoms [7], we found a mastoparan peptide in the Eumenine wasp venom for the first time in 2000. It is eumenine mastoparan-AF (EMP-AF) from Anterhynchium flavomarginatum micado, which has all the chemical and biological characteristics of mastoparans [8,9,10]. EMP-OD (OdVP1) is the second mastoparan to be found in the Eumenine wasp venom of Orancistrocerus drewseni [11,12]. This wasp venom has another mastoparan peptide, OdVP3 [12]. Three other Eumenine wasp venoms have mastoparan peptides: EMP-ER from Eumenes rubrofemoratus, EMP-EF from Eumenes fraterculus, and EpVP2a and EpVP2b from Eumenes pomiformis [13,14]. The sequences of these mastoparan peptides are summarized in Table 1.

Thus, eumenine wasp venoms may have mastoparans in common. The major role of solitary eumenine wasp venom is to paralyze their prey, the caterpillar. Accordingly, these peptides may not contribute to this major role, but have a supporting role in acting as antimicrobials and potentiating the venom toxicity by disturbing excitable membranes, such as similar peptides in spider and scorpion venoms [15].

In our continuing survey of solitary wasp venoms, we found another mastoparan peptide in the venom of *Eumenes micado*. This species is one of the most common Eumenine wasps in Japan. It also belongs to the potter wasps group because they build a potter shaped nest with mud. In this study, we first investigated the LC-MS profile, revealing the major components are mastoparan peptides. Then, they were isolated and their sequences were determined by the conventional method. The results of biological evaluation by using synthetic specimens are also reported.

## 2. Results

### 2.1. On-Line Mass Fingerprinting by LC-MS

**Figure 1:**
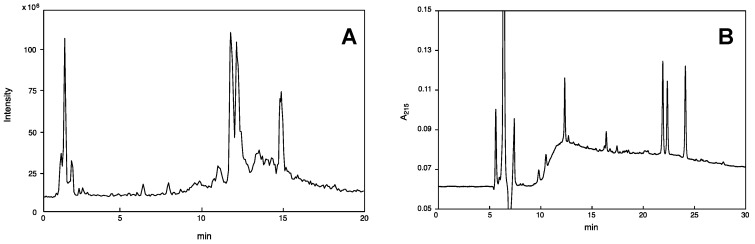
(**A**) TIC profile from LC-ESI-MS of venom extracts of *Eumenes micado*, injected with 10% of crude venom extract of a single specimen to reverse-phase HPLC using CAPCELL PAK C_18_ (1.5 × 150 mm) with linear gradient of 5–65% CH_3_CN/H_2_O/0.1% formic acid over 20 min at flow rate of 200 μL/min. (**B**) Fractionation of venom extracts of *Eumenes micado* by reverse-phase HPLC using CAPCELL PAK C_18_ (10 × 250 mm) with linear gradient of 5–65% CH_3_CN/H_2_O/0.1% TFA over 30 min at flow rate of 2.5 mL/min. UV absorption was monitored at 215 nm.

The crude venom extract was first subjected to LC-ESI-MS in order to know the component profile—how many components are contained and what type of molecules are they. The TIC is shown in Figure 1A. The volume of the sample solution never exceeded 10% amount of crude venom from a single specimen, which is sufficient for LC-ESI-MS analysis (mass fingerprinting and peptide sequencing). On-line mass fingerprint was prepared from TIC by “virtual fractionation”, i.e., collecting MS spectra from certain ranges of retention time, and then the molecular mass was analyzed in each fraction. The results are summarized in Table 2. A total of 93 molecular mass units were found from 18 virtual fractions. The low molecular mass components (*m*/*z* 90–300) may be free amino acids, biogenic amines, and nucleic acids, and those of *m*/*z* range 300–5000 should be peptides, in particular, *m*/*z* 500–2000 accounts for 48%, implying that major components in this venom are relatively small peptides.

### 2.2. Identification of Small Molecules (Amino Acids, Biogenic Amines, and Nucleic Acids)

A total of 25 small molecules (15 amino acids, 4 biogenic amines, 6 nucleic acids) were identified, as summarized in Table 3. It was done mainly by elemental composition analysis of molecular ions (M + H)^+^ with an error limit of 0.005 Da. In some cases for the amino acids, iminium ion was concomitantly observed, analysis of which supported the identification. Concomitant observation of deamination (-NH_3_) peak from molecular ions of biogenic amines was supportive as well. For nucleic acids (adenosine, AMP, ADP, and NAD), MS/MS spectra were obtained by data dependent MS/MS measurement, which confirmed the structure of these compounds.

By conventional HPLC using a reverse-phase C18 column, analysis of these small molecules is not easy because they are eluted as a complex mixture in the front part of the solvent. Accordingly, derivatization or special HPLC conditions are needed for analysis [16,17,18]. In contrast, the method utilizing LC-MS as shown above is easy and advantageous, in that it can be applicable for small molecule analysis of any animal venom.

Previous studies reported the presence and function of some of these components in wasp venoms. Histamine was reported to be found in social and solitary wasp venoms, playing a role in the pain-producing component [16]. Adenosine is contained in spider wasp venom [19]. Dopamine is present in the venom of the emerald jewel wasp Ampulex compressa and implicated in a unique behavior of its prey, the American cockroach [18]. Most of the small molecules contained in this wasp venom would give physiological effects when injected into caterpillar prey, which remains to be studied.

### 2.3. Peptide Sequencing by MS/MS Analysis

Data dependent MS/MS measurement afforded MS/MS spectra from 50 peptide molecules. Manual sequence analysis of these MS/MS spectra revealed the full sequence of 43 peptides, and the rest of the 7 peptides were only partially sequenced (data not shown). The analyzed full sequences are shown in Table 4. The two most intense peaks in Fr. 13 and 14 contained the major peptides EMP-EM1 (*m*/*z* 1481.986, LKLMGLVKKVLGAL-NH_2_: where L = either L or I) and EMP-EM2 (*m*/*z* 1464.032, LKLLGLVKKVLGAL-NH_2_: where L = either L or I), respectively. They are different to each other only at position 4, L vs. M. The second intense peak in Fr. 18 contained two other mastoparan peptides (EMP-EM3: *m*/*z* 1500.940, FDLLGLLKKVVSGL-NH_2_; EMP-EM4: *m*/*z* 1502.902, FDLGMLVKKVLAGL-NH_2_: where L = either L or I).

These sequences can be classified according to homology and similarity. The majority are related to the major peptides EMP-EMs. As shown in Table 5, most of them are truncated peptides from both N- and C-terminus; in other words, they have a partial structure of EMP-EMs. Seemingly, these truncated peptides are cleavage products of EMP-EMs in some way, but it is not certain whether they are originally contained in the venom or not.

The rest of the peptides in this venom may be new peptides, as summarized in Table 6. All of these have no homology to any known peptides.

### 2.4. Purification and Sequence Determination of Mastoparan Peptides

Two major peptides, designated EMP-EM1 and EMP-EM2, were purified by reversed-phase HPLC (Figure 1B) in order to determine their sequences unambiguously. The fractions eluted at 21 and 22 min showed high purity, corresponding to EMP-EM1 and EMP-EM2, respectively. Edman degradation of EMP-EM1 revealed the 13 amino acid sequence as LKLMGIVKKVLGA, and the C-terminal L or I remained undetermined. Accordingly, the two possible structures, LKLMGIVKKVLGAL-NH_2_ and LKLMGIVKKVLGAI-NH_2_, were synthesized by solid-phase method, and the synthetic peptides were compared with natural peptide by HPLC behavior. It clearly showed only the former LKLMGIVKKVLGAL-NH_2_ is identical with the natural one. Similarly, the structure of EMP-EM2 was unambiguously determined as LKLMGIVKKVLGAI-NH_2_. The sequences of two other mastoparan peptides EMP-EM3 and EMP-EM4 were not exactly determined because the HPLC fractionation did not give pure peptides, but instead, an inseparable mixture of these two peptides eluted at 24 min.

**Figure 2:**
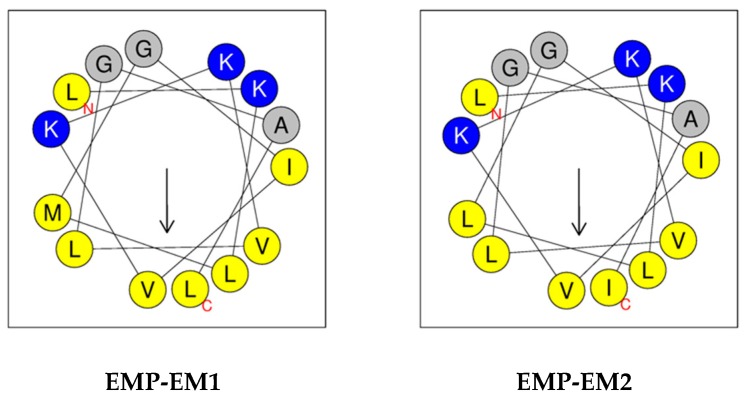
Helical wheel projection of the sequence of EMP-EM1 and EMP-EM2. In this view, through the helix axis, the hydrophilic Lys (K) residues are located on one side and the hydrophobic Val (V), Ile (I), and Leu (L) residues on the other side of the helix.

The chemical features of EMP-EM1 and EMP-EM2, being rich in hydrophobic and basic amino acids with no disulfide bond, are characteristics of mastoparan peptides; in other words, linear cationic α-helical peptides [15]. This class of peptides has been known to adopt an amphipathic α-helical conformation, showing an amphiphilic character under appropriate conditions [10,20,21,22], and the amphipaticity of peptides has been considered essential for their biological activities [23]. In fact, if the helical wheel projection of EMP-EM1 and EMP-EM2 sequences were drawn, amphipathic α-helical conformations could be possible, as depicted in Figure 2. Based on this view, all the hydrophilic amino acid residues (K) are located on one side, whereas the hydrophobic amino acid residues (M, I, L, and V) are on the other side of the helix.

### 2.5. CD Spectroscopy

**Figure 3:**
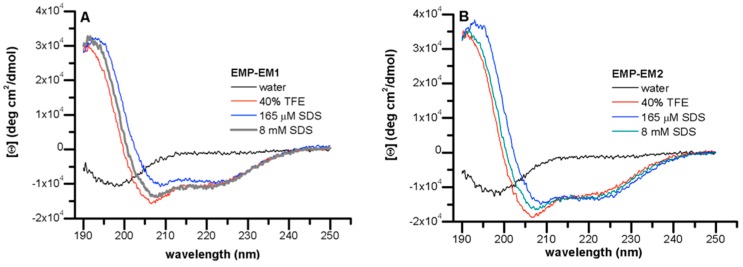
CD spectra of peptides at 10 µM, in different environments, at 25 °C. (**A**) EMP-EM1, (**B**) EMP-EM2.

The mastoparan peptides are known to undergo a conformational change from a random coil to helical upon binding to lipid bilayers or in membrane mimetic environments [9,24]. The α-helix content of these short chain peptides is directly related to favorable electrostatic interactions and the burial of the backbone into a more hydrophobic region. Figure 3 shows the CD spectra of EMP-EM1 and EMP-EM2 obtained in different environments to evaluate the relative importance of the electrostatic and hydrophobic contributions to the observed ellipticity. CD spectra obtained in water and in Tris/borate buffer, pH 7.5, are characteristic of random coil conformation with a dichroic band around 198 nm. In contact with an environment that mimics the anisotropic features of lipid bilayers as SDS suspension above critical micellar concentration (cmc, 8 mM), the CD spectra exhibits two negative bands at 208 and 222 nm and an intense positive band at 192 nm. The spectra were very similar to those obtained in a helical inducer environment of 40% TFE aqueous solution, and even in SDS below the cmc (165 µM) (Figure 3). The ratio between observed ellipticities at 222 nm and 208 nm ([Θ]_222_/[Θ_208_] is <1) is indicative of the presence of the peptides as monomers [25].

Table 7 summarizes CD results and presents physicochemical parameters of EMP-EM1 and EMP-EM2 in comparison to other mastoparan peptides. Except for EMP-AF, all peptides present comparable hydrophobic moments, which is indicative of the amphipaticity of these peptides [26]. EMP-EM1 and EMP-EM-2 present the highest hydrophobicity among these mastoparans, comparable to that observed for EMP-EF and EMP-ER, respectively, while showing no hemolytic activity (shown later). The low helical content observed for EMP-EM1 and EMP-EM2 in the presence of PC vesicles is well correlated with the low hemolytic activity. Accordingly, the higher helical content determined in the presence of anionic SDS solutions indicates the relevance of the electrostatic interactions in the interaction of these peptides with model membranes.

### 2.6. Biological Activities

Biological activities of EMP-EM1 and EMP-EM2 were evaluated by using a synthetic specimen. The mast cell degranulation, hemolysis, antimicrobial, and antiprotozoan (leishmanicidal) activities were tested because these are characteristic biological activities for the mastoparan peptide.

**Figure 4:**
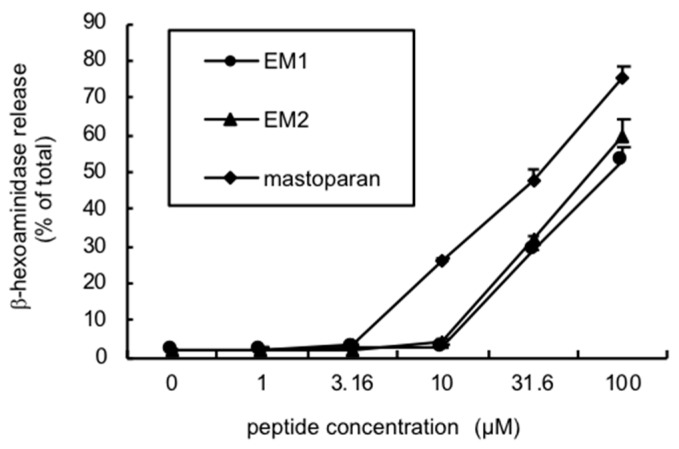
Degranulation activity in rat peritoneal mast cells. The activity was determined by measuring the release of the granule marker, β-hexosaminidase, which co-localizes with histamine, and the values for β-hexosaminidase released in the medium were expressed as the percentage of total β-hexosaminidase, which was determined in the cells lysed in 0.1% Triton X-100.

Both peptides showed a similar potency of degranulation activity on rat peritoneal mast cells, but it was only moderate at a relatively high concentration (>30 μM), and much lower than mastoparan (Figure 4). In contrast, EMP-AF was reported to show more potent activity than mastoparan [8].

The new peptides EMP-EM1 and EMP-EM2 presented no significant hemolytic activity against both human and mouse erythrocytes at the concentration of 10^−4^ M, when comparing with other eumenine mastoparan peptides. Mastoparan itself has an EC_50_ on human erythrocytes of 10^−5^ M [6], while at 10^−4^ M induced 77% hemolysis on mouse erythrocytes. EMP-AF presented 20% of the mastoparan activity, with an EC_50_ of 5 × 10^−5^ M on human erythrocytes [8]. Other eumenine mastoparan peptides (EMP-ER and EMP-EF) presented moderate hemolytic activity on mouse erythrocytes, with EC_50_ values of ~2 × 10^−4^ M [13].

The new mastoparan peptides showed broad spectrum antibacterial activity. The potency is strong to moderate depending on the species and strain, with the lowest MIC being 3 μM. However, they are virtually inactive to yeast (Table 8). This trend is similar to that of EMP-AF [8], but in contrast to EMP-ER and EMP-EF, which had more potent activities against yeast [13].

EMP-EM1 and EMP-EM2 exhibited significant leishmanicidal activity with an IC_50_ of 36 µM against *Leishmania major*, which is comparable to other eumenine mastoparans (Table 9) [13].

## 3. Discussion

In this study, we have first analyzed the component profile of the crude venom of *Eumenes micado*, a solitary eumenine wasp inhabiting Japan, by using LC-MS and MS/MS. It revealed that this venom contained 93 components and that most of them are small peptides. We focused on low molecular weight components in this study because they can be useful for future therapeutic application. That is the reason why the venom components were extracted with 50% CH_3_CN/H_2_O/0.1%. Usually, high molecular weight proteins are not extracted with this condition. However, by using different and appropriate extraction conditions, high molecular weight proteins may be found. In fact, proteins and enzymes, such as arginine kinase, have been found in the venoms of closely related species, *Eumenes pomiformis* and *Orancistrocerus drewseni* [27,28].

The peptide sequences were further analyzed by manual analysis of their MS/MS spectra, which led to the determination of a whole sequence of 50 peptides. Among them, four major peptides were thought to be mastoparan peptides due to the sequence similarity and similarity of characteristic chemical features to mastoparan. Most of the minor peptides are related to, and a truncated form of, the major mastoparan peptides. It is not certain whether they are constitutive of the venom or degradation products of the new mastoparan peptides. In any case, they are of interest from the viewpoint of structure-activity relationship, which may be a future study. Other than these mastoparan-related peptides, only a few peptides shown in Table 6 are unique peptide components in this venom. However, their function and role in this venom are not clear, since they have no homology or similarity to any known peptides. Peptides with disulfide bridges are common in animal venoms, such as snake, spider, and scorpion venoms, and they play a crucial role in the venom toxicity and functions. In the case of solitary wasp venom, the presence of a novel multiple-cysteine peptide with high homology to known venom peptides, dendrotoxin (K+ channel blocker) and Kuniz-type protease inhibitor, was reported [17]. In contrast, there seems no such type of peptides in this wasp venom, which indicates the distribution of disulfide-bridged peptides is different and depends on species or genus, and evolutional origin.

In addition to the peptides, we identified 25 small molecules (amino acids, biogenic amines, and nucleic acids). It was done easily and simply by LC-MS and MS/MS analysis. Since identification of these small molecules are not easy by the conventional HPLC isolation and identification, the method shown in this study is very useful for this purpose. Most notably, these results were obtained by using only 10% of the amount of a single venom content. Among the Hymenopteran insect venoms, solitary wasp venom has not been well-documented. One of the reasons why may come from the difficulty of collecting sufficient amounts of venom for chemical analysis because of their solitary lifestyle. However, as shown in this study, the remarkable progress of mass spectrometry in sensitivity made it possible to perform this type of peptidomic analysis with very minute amount of venom.

With these results in hand, we have purified and characterized the major peptide components, EMP-EM1 and EMP-EM2, by the conventional method. The sequences and chemical characteristics of these peptides are similar to the known mastoparan peptides from solitary eumenine wasp venoms, and accordingly, these new peptides belong to mastoparan peptides; in other words, linear cationic α-helical peptides. Indeed, the CD spectra of these new peptides showed a predominantly α-helix conformation in TFE and SDS.

The biological activities of EMP-EM1 and EMP-EM2 are again similar to those of the known eumenine mastoparan peptides, showing antimicrobial activity and degranulation from mast cells. However, these new peptides showed no significant hemolytic activity to both human and mouse erythrocytes. These results indicated that EMP-EM peptides are strongly associated with bacterial cell membranes rather than mammalian cell membranes. This is in marked contrast to the other eumenine mastoparans, and advantageous for development as therapeutic agents. One of the points of interest for mastoparan peptides is the possible development for a new type of antibiotics due to its potent antimicrobial activity. In this regard, hemolytic activity is a serious drawback as an adverse effect. Accordingly, the new mastoparan peptides found in this study are advantageous and can be a template for further development of new type of antibiotics.

The new mastoparan peptides showed moderate but significant leishmanicidal activity, which is comparable to the known mastoparan peptides from solitary wasp venoms. Leishmaniasis is a parasitic disease caused by protozoal species of the genus Leishmania, and millions of people are afflicted by this disease worldwide. Due to the limitations and drawbacks of the currently available drugs, better, more economical drugs with low toxicity have been long awaited. In this regard, the leishmanicidal activity of the mastoparan peptides is of interest. Since the structure and action mechanism may be quite different from the known drugs, these peptides have a potential as a new type of anti-leishmania agents.

## 4. Materials and Methods

### 4.1. Wasp Collection

Female wasps of *Eumenes micado* were collected in Kanagawa, Ibaraki, and Kyoto, Japan. Only the female wasp has sting and venom because sting is evolved from the ovipositor [29]. The collected specimens were immediately frozen by dry ice and kept at −75 °C until use. The venom sacs were dissected immediately after being thawed and lyophilized.

### 4.2. LC-ESI-MS (Liquid Chromatography-Electrospray Ionization-Mass Spectrometry)

The crude venom extract with 50% CH_3_CN/H_2_O/0.1% TFA was analyzed with a LC (Accela 600 Pump, Thermo Scientific, Waltham, MA, USA) connected with ESI-FTMS (LTQ Orbitrap XL, Thermo Scientific). A 10% amount of crude venom from a single specimen in 10 μL water was subjected to reversed-phase HPLC using CAPCELL PAK C_18_ UG 120, 1.5 × 150 mm (SHISEIDO Co., Ltd., Tokyo, Japan) with linear gradient from 5% to 65% CH_3_CN/H_2_O/0.1% formic acid at a flow rate of 200 μL/min over 20 min at 25 °C. ESI-FTMS was operated by Xcalibar^TM^ software (Thermo Scientific) as: capillary voltage, +4.6 kV; capillary temperature, 350 °C; sheath and aux gas flow, 50 and 30, respectively (arbitrary units). MS/MS spectra were obtained by data dependent MS/MS mode (two most intense peaks by HCD) and the obtained spectra were manually analyzed to give peptide sequences, which were confirmed by MS-Product in the ProteinProspector program (http://prospector.ucsf.edu/prospector/cgi-bin/msform.cgi?form=msproduct).

### 4.3. Purification of Mastoparan Peptides

Twelve lyophilized venom sacs were extracted (5 × 1 mL) with 1: 1 acetonitrile-water containing 0.1% TFA (CH_3_CN/H_2_O/0.1% TFA), and the extracts were subjected to reverse-phase HPLC (Waters Associates, Milford, MA, USA) using CAPCELL PAK C_18_, 10 × 250 mm (SHISEIDO Co., Ltd., Tokyo, Japan) with linear gradient from 5% to 95% CH_3_CN/H_2_O/0.1% TFA at a flow rate of 2.5 mL/min over 30 min. The peaks eluted at 21 and 22 min were manually collected (Figure 1B).

### 4.4. Amino Acid Sequencing

Automated Edman degradation was performed by a gas-phase protein sequencer PPSQ-10 (Shimadzu Corp., Kyoto, Japan).

### 4.5. Peptide Synthesis

Peptides were synthesized on an automated PSSM-8 peptide synthesizer (Shimadzu Corp., Kyoto, Japan) by stepwise solid-phase method using *N*-9-fluorenylmethoxycarbonyl (Fmoc) chemistry. All the resins and Fmoc-L-amino acids were purchased from Nova Biochem (UK). Cleavage of the peptide from the resin was achieved by treatment with a mixture of TFA/1,2-ethanedithiol/thioanisole/phenol/ethyl methyl disulfide/water (82:3:5:3:2:5, by volume) using 10 mL/g resin at room temperature for 8 h. After removal of the resin by filtration and washing twice with TFA, the combined filtrate was added dropwise to diethyl ether at 0 °C and then centrifuged at 3000 rpm for 10 min. Thus, the obtained crude synthetic peptide was purified by semipreparative reverse-phase HPLC using CAPCELL PAK C_18_, 10 × 250 mm with isocratic elution of 42% CH_3_CN/H_2_O/0.1% TFA at a flow rate of 2.5 mL/min. The homogeneity and the sequence were confirmed by LC-MS.

### 4.6. Circular Dichroism (CD) measurements

CD spectra were acquired over 190–250 nm, using a Jasco-710 spectropolarimeter (JASCO International Co. Ltd., Tokyo, Japan) regularly calibrated using d-10-camphorsulfonic acid. Spectra were obtained at 25 °C using 0.5 cm quartz cells in the following environments: in water; in a 40% solution of 2,2,2, trifluoroethanol (40% TFE, from Merck, Darmstadt, Germany); in SDS solutions below (165 µM) and above the critical micellar concentration (8 mM); and in the presence of PC vesicles at 240 µM. Stock solutions of peptides were prepared in ultra-pure water. CD spectra were recorded at 10 µM concentration, at a scan speed of 20 nm/min, bandwidth of 1.0 nm, 0.5 s response, and 0.1 nm resolution. Six repeat scans were accumulated to make up the final averaged spectra. Following baseline correction, the observed ellipticity, θ (mdeg) was converted to mean residue ellipticity [Θ] (deg cm^2^/dmol), using the relationship [Θ] = 100θ/(l c n), where “l” is the path length in centimeters, “c” is peptide millimolar concentration, and “n” the number of residues in the peptide. The α-helix fractions were determined according to the Rohl and Baldwin [30] assuming the two-state model.

### 4.7. Mast Cell Degranulation Activity (β-Hexosaminidase Assay)

Mast cells were obtained by peritoneal lavage of adult (>300 g) Sprague-Dawley rats. The mast cells were isolated from containing cell types by centrifugation through a cushion of Percoll as previously described [31], washed twice by resuspension and centrifugation, and finally suspended in a HEPES buffer, which was comprised of 137 mM NaCl, 2.7 mM KCl, 1 mM MgCl_2_, 1.8 mM CaCl_2_, 20 mM HEPES, 1 mg/mL BSA, and 1 mg/mL glucose (pH 7.4).

Degranulation was determined by measuring the release of the granule marker, N-acetyl-β-d-glucosaminidase (β-hexosaminidase), which co-localizes with histamine, as previously described [31]. The cells were incubated with various concentration of the peptide for 15 min at 37 °C, and then the cells were quenched by addition of 0.15 mL of ice-cold HEPES buffer. After centrifugation, the supernatants were sampled for *β*-hexosamnidase assay. Briefly, 50 mL of samples of the medium and 50 mL of the substrate, 5 mM *p*-nitrophenyl-*N*-acetyl-*β*-d-glucosaminide (Sigma Chemical Co., St. Louis, MO, USA) in 0.2 M citrate, pH 4.5, were incubated in 96-well plates to yield the chromophore, *p*-nitrophenol. The absorbance of the colored product was assessed at 405 nm using a microtiter plate reader. The values for β-hexosaminidase released in the medium were expressed as the percentage of total β-hexosaminidase, which was determined in the cells lysed in 0.1% Triton X-100.

### 4.8. Hemolytic Assay

*Human erythrocytes—*Hemolytic assay was performed as previously described [32] with slight modification. Human blood was obtained from healthy donors at the Vital Brasil Hospital in Butantan Institute. Blood samples drawn to obtain erythrocytes for subsequent use as target cells were collected in anticoagulant (Alsever’s old solution, containing, in mM, 114 citrate, 27 glucose and 72 NaCl, pH 6.1). Erythrocytes were washed three times, resuspended at 2% in PBS, and incubated with sample for 30 min at 37 °C. Background or total cell lysis was evaluated by incubation of erythrocytes with PBS or Triton X-100 (0.5%), respectively. After incubation, unlysed cells were spun down and the absorbance of the supernatant was measured at 414 and 540 nm and expressed as percentage of lysis.

*Mouse erythrocytes—*A 4% suspension of mouse erythrocytes (ES) was prepared as described [33]. Different concentrations of the peptides were incubated with the ES at room temperature (~22 °C) in an Elisa plate (96-wells). After 1 h it was centrifuged (1085 *g*/5 min), and the hemolytic activity of the supernatant was measured by the absorbance at 540 nm, considering as blank the absorbance of Krebs–Henseleit physiological solution (mM: NaCl 113; KH_2_PO_4_ 1.2; KCl 4; MgSO_4_ 1.2; CaCl_2_ 2.5; NaHCO_3_ 25; glucose 11.1), which was the vehicle for the peptides. Total hemolysis was obtained with 1% Triton X-100 and the percentage of hemolysis was calculated relative to this value.

All tests were performed in quadruplicates.

### 4.9. Antimicrobial Activity (Determination of Minimal Inhibitory Concentration, MIC)

The microorganisms used were: *Staphylococcus aureus* (ATCC 6538, ATCC 25923). *S. saprophyticus* (clinical species), *Bacillus subtilis* (CCT 2471), *B. thuringiensis* (wild species), *Echerichia coli* (CCT 1371), *E. coli* (ATCC 25922), *Enterobacter cloacae* (ATCC 23355), *Proteus mirabilis* (clinical species), *Pseudomonas aeruginosa* (ATCC 15442), and *Candida albicans* (UMP).

Müeler-Hinton broth was from Difco. Serial dilution of peptide was prepared in sterilized water. Aliquots were placed in ELISA microplates containing Müeler-Hinton broth in a final volume of 200 μL. The mixture was completed by inoculation of 10 μL of bacterial culture growing in logarithmic-phase of microorganism, as monitored by the UV absorbance at 600 nm. The final cells number (1 × 10^5^/mL) was determined by plate counting.

The plates were incubated at 35 °C and aliquots of 10 μL were removed both at the beginning of assay and after overnight incubation, and then plated in Müeler-Hinton agar. The number of colony-forming units was determined. The results were expressed as inhibition percentage of colony-forming units against a control; this control was obtained in each situation by counting the number of microorganisms introduced into the plate in the absence of peptide.

### 4.10. Leishmanicidal Activity

Medium 199 was used for the cultivation of promastigotes of *Leishmania major* (MHOM/SU/73/5ASKH). Promastigotes were cultured in the medium (supplemented with heat-inactivated (56 °C for 30 min) fetal bovine serum (10%)) at 27 °C, in a 5% CO_2_ atmosphere in an incubator [34].

The leishmanicidal effects of the peptides were assessed using the improved 3-[4,5-dimethylthiazol-2-yl]-2,5-diphenyltetrasodium bromide (MTT) method, as follows. Cultured promastigotes were seeded at 4 × 10^5^/50 μL of the medium per well in 96-well microplates, and then 50 μL of different concentrations of test compounds dissolved in a mixture of DMSO and the medium were added to each well. Each concentration was tested in triplicate. The microplate was incubated at 27 °C in 5% CO_2_ for 48 h. Tetra Color ONE (10 μL) (a mixture of WST-8 (2-(2-methoxy-4-nitrophenyl)-3-(4-nitrophenyl)-5-(2,4-disulfophenyl)-2*H*-tetrazolium, monosodium salt) and 1-methoxy PMS (1-methoxy-5-methylphenazinium methosulfate9)) was added to each well and the plates were incubated at 27 °C for 6 h. Optical density values (test wavelength 450 nm; reference wavelength 630 nm) were measured using a microplate reader (Thermo BioAnalysis Japan Co., Ltd., Kanagawa, Japan). The values of 50% inhibitory concentration of the peptides were estimated from the dose–response curve.

## Figures and Tables

**Table 1:** Mastoparan peptides in wasp venom.

Mastoparan	INLKALAALAKKIL-NH_2_	EMP-AF	INLLKIAKGIIKSL-NH_2_
EMP-EF	FDVMGIIKKIASAL-NH_2_	EMP-OD	GRILSFIKGLAEHL-NH_2_
EMP-ER	FDIMGLIKKVAGAL-NH_2_	OdVP3	KDLHTVVSAILQAL-NH_2_
EMP-EM1	LKLMGIVKKVLGAL-NH_2_	EpVP2a	FDLLGLVKKVASAL-NH_2_
EMP-EM2	LKLLGIVKKVLGAI-NH_2_	EpVP2b	FDLLGLVKSVVSAL-NH_2_

**Table 2:** On-line mass fingerprinting of crude venom extract from *Eumenes micado* by LC-ESI-MS.

Fr. No.	Retention Time (min)	[M + H]^+^ *m*/*z*
1	1.0–1.5	90.054, 106.049, 112.086, 116.070, 118.085, 120.065, 147.076, 147.112, 148.060, 156.076, 175.118, 184.072, 487.358
2	1.5–2.0	132.101, 150.057, 182.080, 268.102, 269.087, 284.097, 317.239, 336.157, 339.237, 348.068, 361.206, 374.324, 418.314, 664.113
3	2.0–3.0	166.085, 428.035
4	3.0–4.0	302.206, 320.162, 359.227, 756.532
5	4.0–5.0	346.232, 372.258, 473.306, 999.663, 1080.504, 2064.020
6	5.0–6.0	599.421, 955.662, 1029.681, 1316.626
7	6.0–7.0	471.326, 543.383, 939.669, 1011.722
8	7.0–8.0	589.331, 883.631, 901.584, 969.639, 2257.190, 2413.290, 3225.613, 4898.1853
9	8.0–9.0	582.256, 931.577, 996.686, 1050.594, 1109.697, 1125.691, 1241.615, 1510.765
10	9.0–10.0	564.299, 1046.653, 1973.176, 2039.289
11	10.0–10.4	940.575, 1074.226, 1449.976, 2607.505
12	10.4–11.4	918.559, 1350.095, 1368.902, 1497.980
13	11.4–12.0	815.497, 1222.854, 1481.986, 1539.990
14	12.0–12.5	1464.032
15	12.5–13.0	2113.272, 2095.261
16	13.0–14.0	1518.894, 2078.259
17	14.0–14.5	1353.871, 1345.833, 1505.866, 3564.943
18	14.5–15.2	1500.941, 1502.903, 1558.943, 1701.020, 1703.984

**Table 3:** Small molecules in the crude venom extract from *Eumenes micado* by LC-ESI-MS.

Retention Time (min)	[M + H]^+^ *m*/*z*
1.10	112.086 (histamine), 147.112 (lysine), 156.076 (histidine)
1.24	175.118 (arginine)
1.31	90.054 (alanine), 106.049 (serine), 116.070 (proline), 118.085 (valine), 120.065 (threonine), 147.076 (glutamine), 148.060 (glutamic acid)
1.53	348.068 (AMP)
1.60	150.057 (methionine), 664.112 (NAD)
1.68	154.085 (dopamine)
1.75	132.101 (leucine/isoleucine), 138.090 (tyramine), 268.102 (adenosine)
1.83	182.080 (tyrosine)
1.98	269.087 (inosine), 284.097 (guanosine)
2.21	428.035 (ADP)
2.44	166.085 (phenylalanine)
2.67	122.096 (phenethylamine)
4.30	205.096 (tryptophan)

**Table 4:** Peptide sequences analyzed from MS/MS spectra.

Fr. No.	[M + H]^+^	Sequence	Fr. No.	[M + H]^+^	Sequence
2	361.206	VVSG	10	564.299	FDLLG
				1046.653	FDLLGLLKK
4	302.206	LLG	12	918.559	FDLGLLK
	320.162	LMG		1350.095	KLLGLVKKVLGAL-NH_2_
	359.227	VLQ		1368.902	KLMGLVKKVLGAL-NH_2_
	756.532	LVKKVLG		1497.980	LKLmGLVKKVLGAL-NH_2_
5	346.232 372.258 473.306	LLT LGAL-NH_2_ VVSGL-NH_2_	13	815.497 1222.852 1481.986 1539.990	PVGFLGLL LLGLSLVLLGLLL-NH_2_ LKLMGLVKKVLGAL-NH_2_ LKLMGLVKKVLGALG
6	599.421	KVLGAL-NH_2_	14	1464.032	LKLGLVKKVLGAL-NH_2_
	955.662	LLKKVVGSL-NH_2_			
	1029.681	LKKMGLVKK			
7	471.326	VLGAL-NH2	16	1518.894	FDLGmLVKKVLAGL-NH_2_
	543.383	LKLLG			
	939.669	LVKQKVLL-NH_2_			
	1011.722	LKLLGLVKK			
8	589.331	PVGFLG	17	1353.871 1355.833	DLLGLLKKVVSGL-NH_2_ DLGMLVKKVLAGL-NH_2_
	883.631	LKLLGLVK			
	901.584	LKLMGLVK			
	969.639	LNLLKLAKG			
9	582.256	FDLGM	18	1500.941 1502.903 1558.943	FDLLGLLKKVVSGL-NH_2_ FDLGMLVKKVLAGL-NH_2_ FDLLGLLKKVVSGLG
	931.577	TLKVGSLLT			
	1109.697	VLNVLNVLL-NH_2_			
	1125.691	VLNTQNVLL-NH_2_			
	1241.615	LKLMGLVKKVL			

L = either L or I; m = methionine S-oxide.

**Table 5:** Peptides related to EMP-EMs.

RT	[M + H]^+^	Sequence
**EMP-EM1**		
7.16	901.584	LKLMGLVK
6.02	1029.681	LKLMGLVKK
8.81	1241.615	LKLMGLVKKVL
1.88	336.157	LmG
3.09	320.162	LMG
3.32	756.532	LVKKVLG
4.94	372.259	LGAL-NH_2_
6.55	471.326	VLGAL-NH_2_
5.12	599.420	KVLGAL-NH_2_
10.65	1368.906	KLMGLVKKVLGAL-NH_2_
10.64	1497.980	LKLmGLVKKVLGAL-NH_2_
11.72	1481.987	LKLMGLVKKVLGAL-NH_2_ (EMP-EM1)
11.59	1539.995	LKLMGLVKKVLGALG
**EMP-EM2**		
6.80	543.883	LKLLG
7.64	883.631	LKLLGLVK
6.47	1011.722	LKLLGLVKK
3.63	302.206	LLG
10.84	1350.095	KLLGLVKKVLGAL-NH_2_
12.12	1464.032	LKLLGLVKKVLGAL-NH_2_ (EMP-EM2)
**EMP-EM3**		
9.25	564.299	FDLLG
10.77	918.559	FDLLGLLK
9.54	1046.653	FDLLGLLKK
1.73	361.206	VVSG
4.73	473.305	VVSGL-NH_2_
5.35	955.662	LLKKVVSGL-NH_2_
14.42	1353.871	DLLGLLKKVVSGL-NH_2_
14.76	1500.940	FDLLGLLKKVVSGL-NH_2_ (EMP-EM3)
14.57	1558.951	FDLLGLLKKVVSGLG
**EMP-EM4**		
8.83	582.256	FDLGM
14.43	1355.833	DLGMLVKKVVSGL-NH_2_
13.50	1518.895	FDLGmLVKKVVSGL-NH_2_
14.89	1502.902	FDLGMLVKKVVSGL-NH_2_ (EMP-EM4)

L = either L or I; m = methionine S-oxide.

**Table 6:** Unknown peptides.

RT	[M + H]^+^	Sequence
8.58	931.575	TLKVGSLLT
8.51	1125.691	VLNTQLNVLL-NH_2_
8.67	1109.697	VLNVNLNVLL-NH_2_
7.91	589.331	PVGFLG
11.89	815.498	PVGFLGLL
11.59	1222.851	LLGLSLVLGLLL-NH_2_

L = either L or I; m = methionine S-oxide.

**Table 7:** Physicochemical parameters and α-helical content in different environments of EMP-EM1 and EMP-EM2 in comparison to other mastoparan peptides.

Peptides	N	Q	C-term	<H>	*µ*	*f_H_*		
						TFE	SDS	PC
**EMP-EM1**	14	+4	amide	0.104	0.258	0.31	0.33	0.11
**EMP-EM2**	14	+4	amide	0.138	0.278	0.37	0.41	<0.03
**EMP-AF^1^**	14	+4	amide	0.051	0.342	0.55	0.72	0.16
**EMP-ER^2^**	14	+2	amide	0.131	0.251	0.53	0.59	nd
**EMP-EF^2^**	14	+2	amide	0.115	0.279	0.41	0.44	nd

N, number of residues; Q, net charge; C-term, C-terminal; <H>, mean hydrophobicity; *μ*, hydrophobic moment; ***f*_H_**, α-helix fraction: 40% TFE, 8 mM SDS; 380 µM PC; nd, non-determined. (1) dos Santos Cabrera et al., 2004 [9]; (2) Rangel et al., 2011 [13].

**Table 8:** Antimicrobial activity of EMP peptides.

Microorganism	MIC (µM) *	
	EM1	EM2
**Gram-positive**		
*Staphylococcus aureus* ATCC 6538	34	17
*Staphylococcus aureus* ATCC 25923	7	3
*Staphylococcus saprophyticus* (CS)	3	3
*Staphylococcus epidermis* (CS)	34	34
*Bacillus subtilis* CCT 2471	68	68
*Bacillus thuringiensis* (WT)	NA**	NA
**Gram-negative**		
*Escherichia coli* ATCC 25922	7	3
*Escherichia coli* CCT 1371	17	34
*Escherichia cloacae* ATCC 23355	17	34
*Proteus mirabilis* (CS)	NA	NA
*Pseudomonas aeruginosa* ATCC 15442	NA	34
**Yeast**		
*Candida albicans* (UMP)	NA	NA

Note: * MIC: minimum inhibitory concentration. ** NA: no activity at 67 or 68 μM (100 μg/mL).

**Table 9:** Leishmanicidal activity of the mastoparan peptides.

Peptide	IC50 (μM) *
EMP-EM1	36
EMP-EM2	36
EMP-ER	20 **
EMP-EF	40 **
EMP-AF	35 **

Note: * IC_50_: 50% inhibitory concentration. ** Rangel. M. et al. [11].
